# A novel optical sign in keratoconus: the Halo Sign and its clinical characteristics

**DOI:** 10.1186/s12886-026-04718-8

**Published:** 2026-03-14

**Authors:** Hui He, Shiyang Niu, Lijun Qu

**Affiliations:** Department of Ophthalmology, The Second Affliated Hospital of Harbin Medical University, No. 246 Xuefu Road, Nangang District, Harbin, 150086 China

**Keywords:** Keratoconus, Halo Sign, Slit-lamp, Disease severity

## Abstract

**Background:**

The Halo sign is a recently identified slit-lamp optical phenomenon observed in keratoconus. This study aims to systematically characterize this phenomenon and investigate its clinical features in eyes with keratoconus.

**Methods:**

This retrospective, observational case series included 11 consecutive patients (22 eyes) with a clinical diagnosis of KC from a single tertiary center. Slit-lamp biomicroscopy with the beam directed at the cone apex was performed to evaluate the presence of the Halo Sign. A quantitative Halo Morphology Index (HMI) was defined as the aspect ratio (major/minor axis; a/b) of an ellipse fitted to the segmented halo outline on slit-lamp photographs and measured in ImageJ. Clinical and imaging characteristics were summarized descriptively, including corrected distance visual acuity (CDVA, logMAR), Amsler-Krumeich (AK) stage, thinnest corneal thickness (Thk), keratometric indices (e.g., mean corneal refractive power and mean pupillary power), keratoconus vertex distance (KVD), and corneal higher-order aberrations (HOAs). AS-OCT structural staging was recorded when gradable.

**Results:**

The Halo Sign was absent in 14 eyes and present in 8 eyes. Halo-positive eyes were predominantly advanced by AK staging (all 8 eyes were AK stage 4), whereas Halo-negative eyes spanned a wider range of AK stages (1–4). In descriptive comparisons, Halo-positive eyes tended to have worse CDVA (logMAR), thinner corneas, steeper keratometry, and a higher prevalence of corneal scarring than Halo-negative eyes. Cone decentration (KVD) was also greater in Halo-positive eyes. AS-OCT structural staging was available in 19/22 eyes, with Halo-positive eyes more frequently classified as Sandali OCT stage 2 than Halo-negative eyes. HMI values in Halo-positive eyes ranged from 1.07 to 3.93, reflecting increasing halo outline anisotropy/irregularity.

**Conclusions:**

The Halo Sign is a newly described slit-lamp optical phenomenon that was observed predominantly in advanced keratoconus in this exploratory case series. HMI provides an operational, image-based descriptor of halo outline anisotropy.

## Introduction

Keratoconus (KC) is an asymmetric, progressive corneal ectatic disorder that typically manifests during adolescence [1]. Its etiology is not yet fully understood and is currently considered to be multifactorial, potentially involving genetic predisposition, environmental factors, and eye rubbing [2–5]. It is characterized by progressive localized corneal thinning and protrusion, leading to high myopia and astigmatism, which can result in severe visual impairment and even blindness [6, 7]. Epidemiological studies indicate a vast disparity in the global prevalence of KC, with estimates ranging from 0.2 to 4,790 cases per 100,000 individuals. The estimated range for its annual incidence is similarly wide, reported as 1.5 to 25 cases per 100,000 individuals [2, 8, 9]. The significant variation in reported global prevalence rates may be attributed to differences in diagnostic methods and criteria, the racial or ethnic composition of patient populations, age, and other factors. Therefore, establishing more comprehensive diagnostic strategies to improve the detection rate of this disease holds significant clinical and public health importance.

The current diagnosis and staging of KC primarily rely on clinical signs, morphological indices, and biomechanical parameters [10–16]. In resource-limited settings where equipment such as corneal topography and biomechanical assessments are unaffordable, clinical signs become the primary basis for clinicians to diagnose KC. Over the past decades, several classic slit-lamp signs of KC have been described, such as Vogt’s striae and Fleischer’s rings. Recently, He and Qu (2025) [17] described a novel optical phenomenon in advanced KC, termed the “Halo Sign,” which manifests as an annular light reflex on the iris when the slit-lamp beam illuminates the cone apex. Classic slit-lamp signs such as Vogt’s striae primarily reflect alterations in corneal biomechanical stress, whereas Fleischer’s ring represents epithelial iron deposition. In contrast, the Halo Sign appears to be predominantly an optical phenomenon. This finding suggests that optical alterations in the ectatic cornea may contribute to the formation of a clinically observable sign.

The characterization of this Halo Sign, however, remains preliminary. While its presence has been noted, whether its morphological features, such as shape, completeness, and regularity, correlate with the quantitative severity of KC has not been further investigated.

## Materials and methods

### Study design and participants

This retrospective, observational case series was approved by the Ethics Committee of The Second Affiliated Hospital of Harbin Medical University (Approval No: KY2024-199) and adhered to the tenets of the Declaration of Helsinki. Written informed consent was obtained from all participants.

We consecutively enrolled 11 patients (22 eyes) with a clinical diagnosis of keratoconus (KC) presenting to our clinic between October 2023 and July 2025. Because the Halo Sign has only recently been described and reliable prior effect-size estimates were not available, we did not prespecify an a priori sample size. Instead, we included all consecutive eligible patients/eyes presenting during a predefined study period, which helps minimize selection bias within this retrospective design. Given that keratoconus is a bilateral but typically asymmetric ectatic disorder, both eyes of each patient were included in the analysis. Previous studies have demonstrated significant inter-eye differences in morphological and tomographic parameters, and such asymmetry has been associated with disease severity and progression, supporting the rationale for analyzing each eye individually [18]. The diagnosis of keratoconus was established when abnormal topographic or tomographic findings were accompanied by at least one clinical sign [1, 19]. Exclusion criteria included a history of ocular trauma or surgery, active keratitis, glaucoma, or significant iris abnormalities that could interfere with halo visualization. Eyes with KC were classified into two groups according to halo imaging characteristics: Group A, without halo formation under multi-angle corneal illumination, and Group B, with regular or irregular halo formation.

### Clinical examination and imaging protocol

All participants underwent a comprehensive ophthalmic examination, including corrected distance visual acuity (CDVA). Standardized digital slit-lamp images were captured for all eyes using a biomicroscope (Shanghai MediWorks Precision Instruments Co., Ltd., Shanghai, China). Corneal tomographic parameters were acquired using a Scheimpflug-Placido topographer (Sirius, Phoenix software v.2.6.4.44, Costruzione Strumenti Oftalmici, Florence, Italy). For each eye, three consecutive scans were performed, and the average values were used for analysis. Wavefront aberrations were calculated for the anterior corneal surface using a standardized 3.0-mm analysis diameter. Aberration values were expressed as root mean square (RMS) values and derived from Zernike polynomial expansion up to the 6th order, as implemented in the instrument’s software. High-resolution images of the corneal layers were obtained using Anterior Segment Optical Coherence Tomography (AS-OCT) (RTVue XR Avanti; Optovue, Inc., Fremont, CA, USA).

### Measurement of the Halo Sign and Halo Morphology Index

**Fig. 1:**
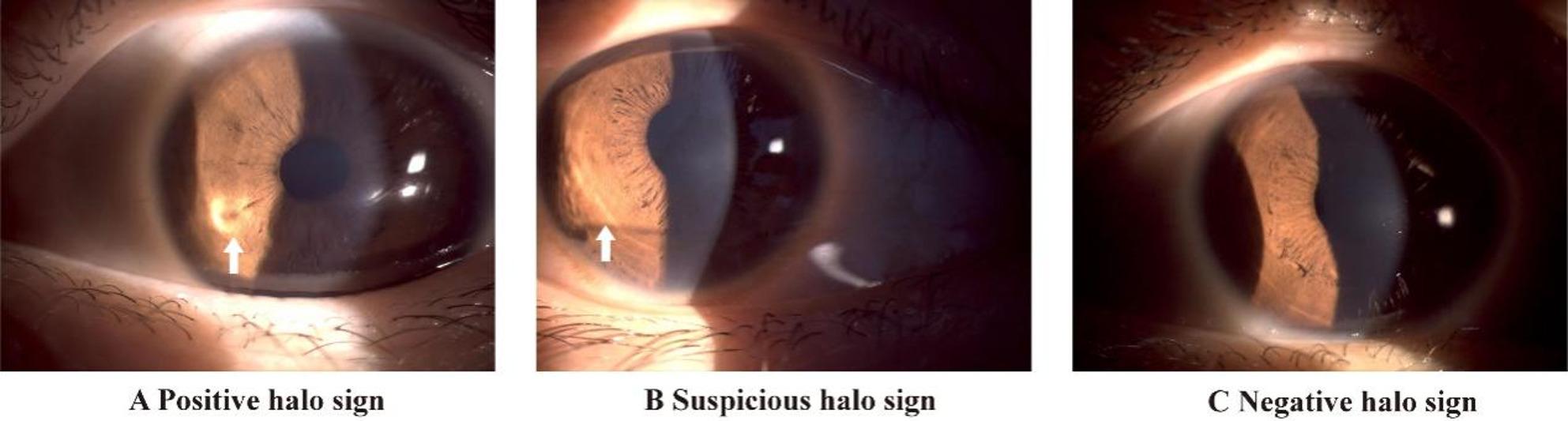
Identification of the Halo Sign. **A**, Positive Halo sign: a clearly identifiable continuous annular light projection on the iris. **B**, Suspicious Halo sign: localized increase in iris luminance without formation of a continuous annular projection. **C**, Negative Halo sign: uniform light distribution on the iris with no observable annular structure

Halo signs were examined using a slit-lamp biomicroscope. Participants were seated with the chin and forehead stabilized on the headrest and instructed to maintain primary gaze at a distant fixation target. Examinations were performed under natural pupil conditions without pharmacologic dilation. Ambient illumination was reduced to enhance contrast. A narrow slit beam was first used to obtain an optical section of the cornea to identify the point of maximal anterior protrusion. The slit height was then adjusted to fully cover the corneal diameter, and the beam width was modified as needed to precisely illuminate this location. The light beam was delivered with variable slit widths and an incident angle ranging from 0° to 80°. Observations were conducted at 10× magnification under a standard anterior segment working distance (approximately 50–100 mm for the MediWorks slit-lamp system). Subsequently, the focal plane was shifted to the iris plane to optimize visualization of the projected Halo, and the presence or absence of the Halo sign was recorded (Fig. 1).

Two independent ophthalmologists, masked to tomographic data, evaluated anonymized and randomized slit-lamp photographs for the presence and morphology of the Halo Sign. For quantitative HMI measurement, both graders completed the full ImageJ workflow independently. To assess intra-observer repeatability, each grader repeated measurements after a 2-week washout period (grader 1 ICC = 0.995, 95% CI: 0.976–0.999; grader 2 ICC = 0.918, 95% CI: 0.612–0.981). Inter-observer reproducibility was assessed using the two graders’ first measurements (ICC = 0.957, 95% CI: 0.812–0.992).

### Halo Morphology Index (HMI): definition and interpretation

HMI was defined as the ellipse aspect ratio of the halo outline on the iris: HMI = AR = a/b, where a and b are the major and minor axes of the best-fit ellipse (a ≥ b). In ImageJ, AR was obtained by enabling “Fit ellipse” and “Shape descriptors” (Analyze > Set Measurements) and recording the AR reported by Analyze > Measure (equivalent to Major/Minor). Clinically, values close to 1 indicate a near-circular halo outline, whereas larger values indicate greater outline elongation (eccentricity), used here as an operational measure of halo outline shape anisotropy. Eyes without a detectable halo were assigned HMI = 0 by definition. Importantly, HMI does not directly quantify ring completeness (e.g., circumferential coverage or gap size).

### Image-processing and segmentation workflow (ImageJ)

**Fig. 2:**
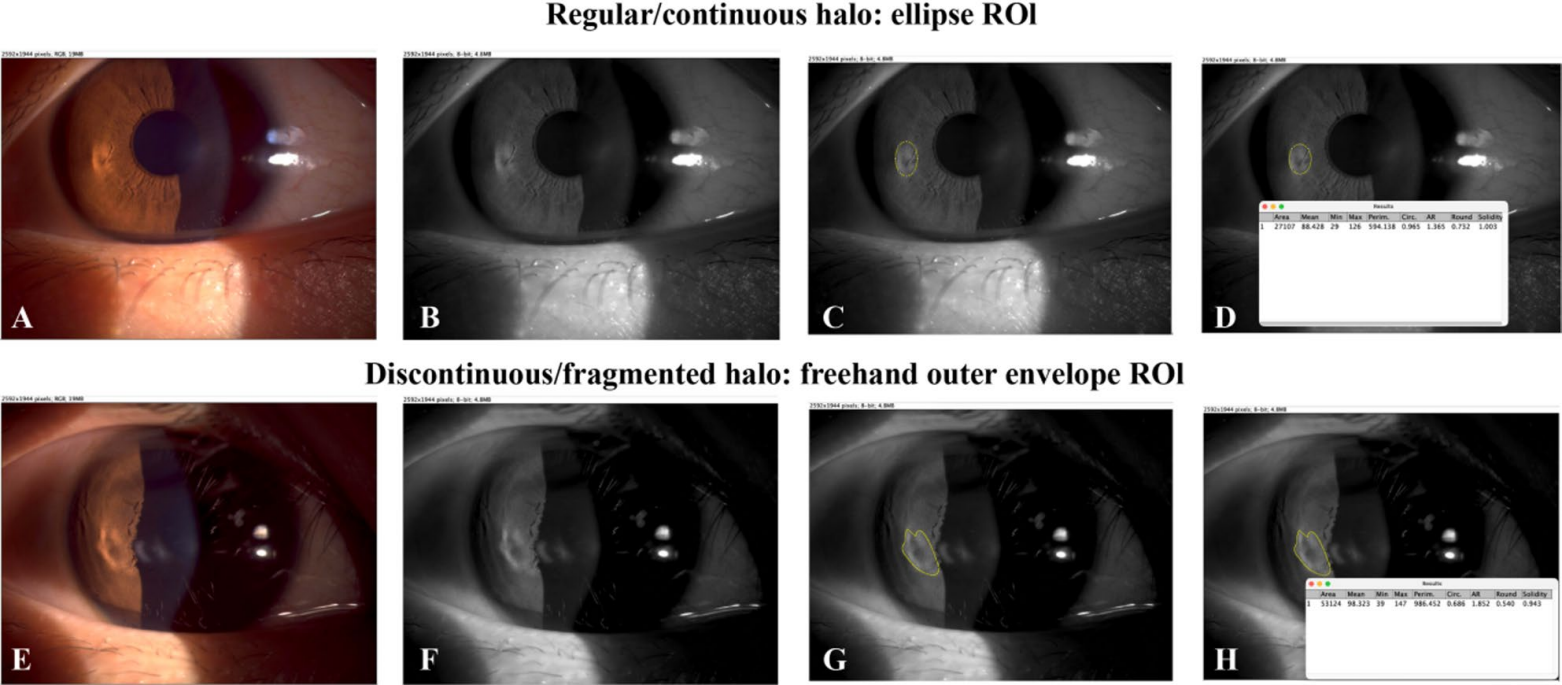
ImageJ workflow for HMI measurement. Top row (**A**–**D**): regular/continuous halo measured using an elliptical ROI fitted to the halo outer boundary. Bottom row (**E**–**H**): discontinuous/fragmented halo measured by freehand tracing of the outer envelope of all visible halo segments. In each row: (**A**/**E**) original slit-lamp photograph; (**B**/**F**) green-channel image after RGB splitting; (**C**/**G**) ROI delineation of halo outer boundary/envelope while avoiding specular highlights and eyelid/lash shadows; (**D**/**H**) ImageJ measurement output after enabling “Fit ellipse” and “Shape descriptors,” where AR (Major/Minor) is recorded as HMI

Slit-lamp photographs were quantified in ImageJ (NIH, USA) using a standardized, rater-masked, manual ROI workflow with overlay verification. Each RGB image was split into channels (Image > Color > Split Channels); the green channel was analyzed because it provided the most consistent halo–background contrast. For visualization only, linear brightness/contrast adjustment and zooming were allowed; no geometric scaling, warping, or resampling was performed. Because illumination and halo intensity varied across retrospective photographs, no automated thresholding was applied. Segmentation targeted the outer boundary/envelope of the visible halo: for regular, continuous halos, an elliptical ROI was positioned to best match the halo’s outer boundary; for discontinuous, faint, or fragmented halos, the outer envelope of all visible segments was traced using the freehand selection tool, avoiding specular highlights and eyelid/lash shadows. If a halo boundary could not be reliably identified, the eye was classified as “no detectable halo” (HMI = 0) and was not quantified. The final ROI was saved in the ROI Manager and overlaid on the original image for documentation (Fig. 2).

### Data collection and outcome measures

The primary focus of this study was to document the clinical features and morphological patterns of the Halo Sign, and to describe how HMI varied across KC phenotypes and key clinical/tomographic characteristics. Clinical and tomographic assessments were performed. Corrected Distance Visual Acuity (CDVA) was recorded, and the presence of corneal scarring was evaluated via slit-lamp biomicroscopy. Key tomographic parameters were extracted from the Sirius topographer scans for each eye. These included: Thinnest Corneal Thickness (Thk), Mean Pupillary Power (MPP), Keratoconus Vertex Distance (KVD), curvature at the thinnest point (Thk point curvature), mean corneal refractive power (Km), total Higher-Order Aberrations (HOA), and Coma.

Keratoconus severity was graded using the Amsler–Krumeich (AK) classification [20], operationalized using objective criteria available in this retrospective dataset. Anterior segment OCT images were reviewed and staged using the structural classification proposed by Sandali et al. [21]. High-resolution corneal line/cross-line scans passing through the cone apex were used for grading when available. OCT staging was feasible in 19 of 22 eyes; in three eyes, AS-OCT images were not gradable because adequate scans could not be obtained (unmeasurable scans and/or poor cooperation), and OCT stage was recorded as missing for descriptive reporting.

### Statistical analysis

Statistical analyses were descriptive, consistent with the exploratory nature of this retrospective case series. Continuous variables are summarized as mean ± standard deviation (SD), and categorical variables as number (percentage). No formal hypothesis testing, regression modeling, or multiple-comparison adjustment was performed. The measurement reliability of the Halo Morphology Index (HMI) was assessed using the intraclass correlation coefficient (ICC(2,1)) with 95% confidence intervals (CI). No a priori sample size was prespecified because the Halo Sign is newly described and reliable prior effect-size estimates were not available; therefore, the sample size was determined by the number of consecutive eligible patients/eyes available during the predefined study period.

## Results

### Baseline demographic and clinical characteristics

A total of 22 eyes from 11 patients with keratoconus (7 males and 4 females) were included. The mean age at the time of definitive diagnosis was 25.00 ± 6.54 years, with a range of 14 to 37 years. Detailed demographic, clinical, and keratoconus parameter characteristics for all subjects are presented in Table 1.

**Table 1:** Demographic, clinical, and KC parameter characteristics by group

Group	case	sex	age	eye	HMI	AK Stage	OCT stage	CDVA (LogMAR)	Thk(µm)	MPP(D)	KVD(µm)	Thk Point Curvature (D)	Km (D)	HOA (µm)	Coma	Scar
Group A	case 1	M	27	R	0	1	1	0.40	424	45.02	81	64.75	46.94	1.46	1.29	-
				L	0	1	1	0.10	419	44.72	66	60.53	45.80	1.41	0.89	-
	case 2	F	14	R	0	4	1	0.70	401	53.77	137	73.27	55.32	2.6	2.36	-
				L	0	3	1	0.70	430	50.73	96	66.04	53.20	2.39	1.63	-
	case 3	M	21	R	0	1	1	0.10	483	44.93	16	48.18	45.50	0.54	0.1	-
				L	0	3	1	0.40	395	46.86	158	72.88	53.54	3.06	1.13	-
	case 4	M	23	R	0	1	1	0.10	502	41.49	13	43.62	42.05	0.34	0.01	-
	case 5	F	30	R	0	1	1	0.10	467	44.79	40	51.60	45.59	2.68	0.63	-
	case 6	M	25	L	0	1	1	0.22	488	45.73	40	49.65	45.85	1.13	0.18	-
	case 7	F	37	R	0	1	1	0.10	497	46.17	42	51.91	46.14	1.78	0.56	-
	case 8	F	27	R	0	3	-	0.70	366	47.62	55	57.20	48.47	2.02	0.55	-
	case 9	M	19	R	0	4	1	1.00	314	69.11	295	84.22	69.43	2.29	1.31	-
	case 10	M	20	R	0	1	1	0.05	514	43.14	11	45.78	44.05	0.89	0.12	-
	case 11	M	32	R	0	1	1	0.05	521	42.46	13	44.26	43.04	0.43	0.03	-
Group B	case 4	M	23	L	1.36	4	2	1.00	420	51.26	208	79.85	55.49	4.07	2.46	-
	case 5	F	30	L	1.07	4	2	1.00	363	58.08	247	103.65	59.55	11.93	10.05	-
	case 6	M	25	R	1.85	4	2	1.00	360	69.79	286	123.3	60.44	7.6	3.47	+
	case 7	F	37	L	3.34	4	2	2.30	328	78.07	299	107.65	68	10.36	4.22	+
	case 8	F	27	L	3.55	4	-	1.00	252	80.08	327	88.16	76.66	2.91	2.17	+
	case 9	M	19	L	3.53	4	2	1.00	314	69.11	295	84.22	69.43	2.29	1.31	+
	case 10	M	20	L	3.93	4	2	1.00	414	48.43	58	61.46	46.12	1.45	0.91	+
	case 11	M	32	L	3.48	4	-	2.30	unable to be detected							++

Note: HMI: Halo Morphology Index; CDVA: Corrected Distance Visual Acuity; Thk: Thinnest Corneal Thickness; MPP: Mean Pupillary Power; KVD: Keratoconus Vertex Distance; Km: Mean Keratometry; HOA: High Order Aberration; M/F: Male/Female; R/L: Right Eye/Left Eye; Scarring - clear, no scarring (-), scarring, iris details visible (+), scarring, iris obscured (++)

### Characteristics of the Halo Sign

**Fig. 3:**
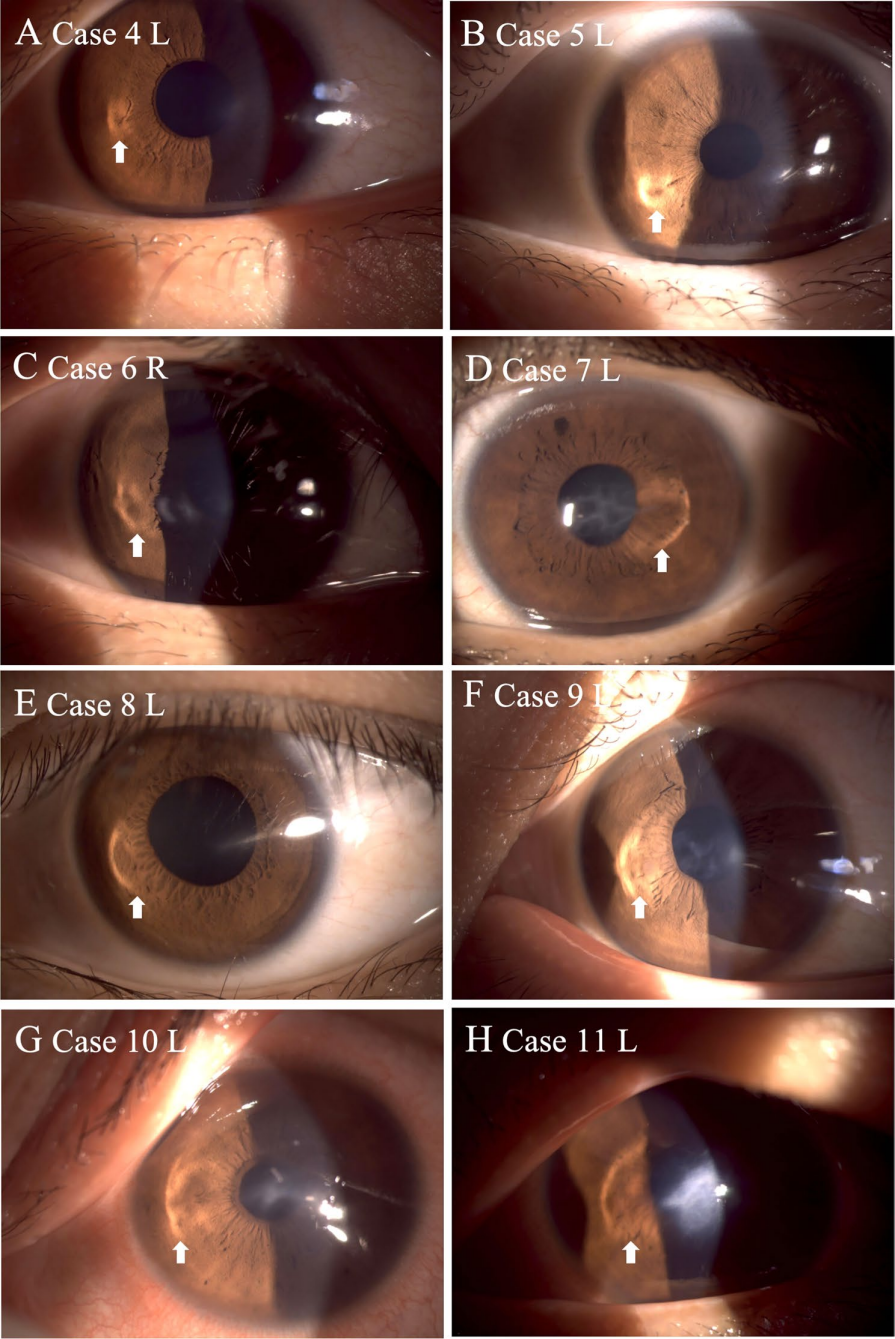
Morphological spectrum of the Halo Sign in keratoconus patients. This figure displays anterior segment photographs of 8 eyes exhibiting the Halo Sign, labeled (**A**–**H**) to illustrate the progression from regular to irregular morphologies. White arrowheads indicate the location of the Halo Sign. (**A**, **B**) Cases 4 and 5 present a continuous, well-defined annular halo with relatively regular geometry, corresponding to lower Halo Morphology Index (HMI) values (1.36 and 1.07, respectively). (**C**) Case 6 exhibits a recognizable halo but with minor local irregularities at the border (HMI = 1.85). (**D**–**H**) Cases 7 through 11 demonstrate severe morphological distortion where the halo has completely lost its annular integrity, appearing as irregular, fragmented spots. These cases correspond to significantly higher HMI values (ranging from 3.34 to 3.93), highlighting the correspondence between elevated HMI and increased morphological irregularity

Based on slit-lamp examination, the Halo Sign was absent in 14 eyes (Group A) and present in 8 eyes (Group B), exhibiting varying degrees of morphological regularity (Fig. 3). The Halo Sign was observed exclusively in AK stage IV eyes in this series (8/8 Halo-positive eyes), although a small subset of AK stage IV eyes did not exhibit a detectable halo (2/14 Halo-negative eyes). The morphology of the sign varied. The quantitative HMI (AR) for Group B ranged from 1.07 to 3.93 (Table 1). In this cohort, measurable HMI values were observed only in advanced-stage eyes, indicating that higher HMI reflects halo restriction to advanced disease in this sample.

Compared with Group A, eyes in Group B showed worse clinical status and more severe tomographic features. Clinically, Group B tended to have worse CDVA and a higher prevalence of corneal scarring. Regarding tomographic parameters, Group B generally exhibited reduced thinnest pachymetry with increased keratometric indices (e.g., MPP, KVD, thinnest-point curvature, and Km). HOA and coma also tended to be higher in Group B (Table 2).

**Table 2:** Demographics and clinical characteristics of study groups

Parameters	Group A (*n* = 14)	Group B (*n* = 8)
Demographics		
Age (years)	24.07 ± 6.55	26.62 ± 6.16
Male Sex, n (%)	9 (64.3%)	5 (62.5%)
Clinical Parameters		
Corneal Scarring, n (%)	0 (0.0%)	6 (75.0%)
CDVA (LogMAR)	0.34 ± 0.32	1.33 ± 0.60
AK Stage	1.86 ± 1.23	4.00 ± 0.00
OCT Stage	1.00 ± 0.00	2.00 ± 0.00
Thk (µm)	444.36 ± 61.67	350.14 ± 58.66
MPP (D)	47.61 ± 6.96	64.97 ± 12.58
KVD (µm)	75.93 ± 77.90	245.71 ± 91.43
Thk Point Curvature (D)	58.14 ± 12.55	92.61 ± 20.47
Km (D)	48.92 ± 7.13	62.24 ± 10.07
HOA (µm)	1.64 ± 0.90	5.80 ± 4.17
Coma	0.77 ± 0.70	3.51 ± 3.10

Note: CDVA: Corrected Distance Visual Acuity; Thk: Thinnest Corneal Thickness; MPP: Mean Pupillary Power; KVD: Keratoconus Vertex Distance; Km: Mean Keratometry; HOA: High Order Aberration. Data are presented as Mean ± Standard Deviation for continuous variables, and number (percentage) for categorical variables. OCT stage summaries are based on gradable AS-OCT scans (Group A: *n* = 13; Group B: *n* = 6)

### Disease severity by AK and AS-OCT structural staging

By Amsler–Krumeich (AK) staging, all Halo-positive eyes were classified as stage IV (8/8), whereas Halo-negative eyes spanned stages I–IV (stage I: 9/14; stage III: 3/14; stage IV: 2/14) (Tables 1 and 2). AS-OCT structural staging (Sandali classification) was feasible in 19/22 eyes; among gradable scans, Halo-positive eyes were more frequently classified as OCT stage 2 (6/6), whereas Halo-negative eyes were classified as OCT stage 1 (13/13).

### Descriptive patterns of HMI across KC characteristics

Descriptively, higher HMI values tended to co-occur with worse structural severity, including thinner corneas and steeper keratometry, consistent with the stage-stratified summaries. Given the small and imbalanced sample and confounding by severity and scarring, these observations are hypothesis-generating rather than confirmatory.

### Representative case presentations

To illustrate the clinical spectrum of the Halo Sign, representative cases from each group are presented.

### Case 1 R: keratoconus without halo formation (Group A, HMI = 0; Fig. 4)

A 27-year-old male presented with a clinical diagnosis of stage 1 keratoconus. The patient reported progressive vision loss in the right eye over the past 8 months. He had a history of wearing frame glasses and had not previously undergone any other form of refractive correction. Ophthalmic examination revealed an uncorrected distance visual acuity (UDVA) of 0.52. Manifest refraction indicated compound myopic astigmatism (-2.75 DS / -4.75 DC × 7°), achieving a corrected distance visual acuity (CDVA) of 0.4. Slit-lamp examination revealed a Fleischer’s ring without corneal scarring. Under multi-angle slit illumination, no annular halo was formed on the iris. AS-OCT showed mild posterior corneal protrusion with preserved stromal architecture. The tangential curvature map demonstrated localized inferocentral steepening, while the HOA map showed vertical coma with moderate asymmetry. This early-stage case exemplifies our observation that halo absence was more frequently seen in earlier-stage eyes.

**Fig. 4:**
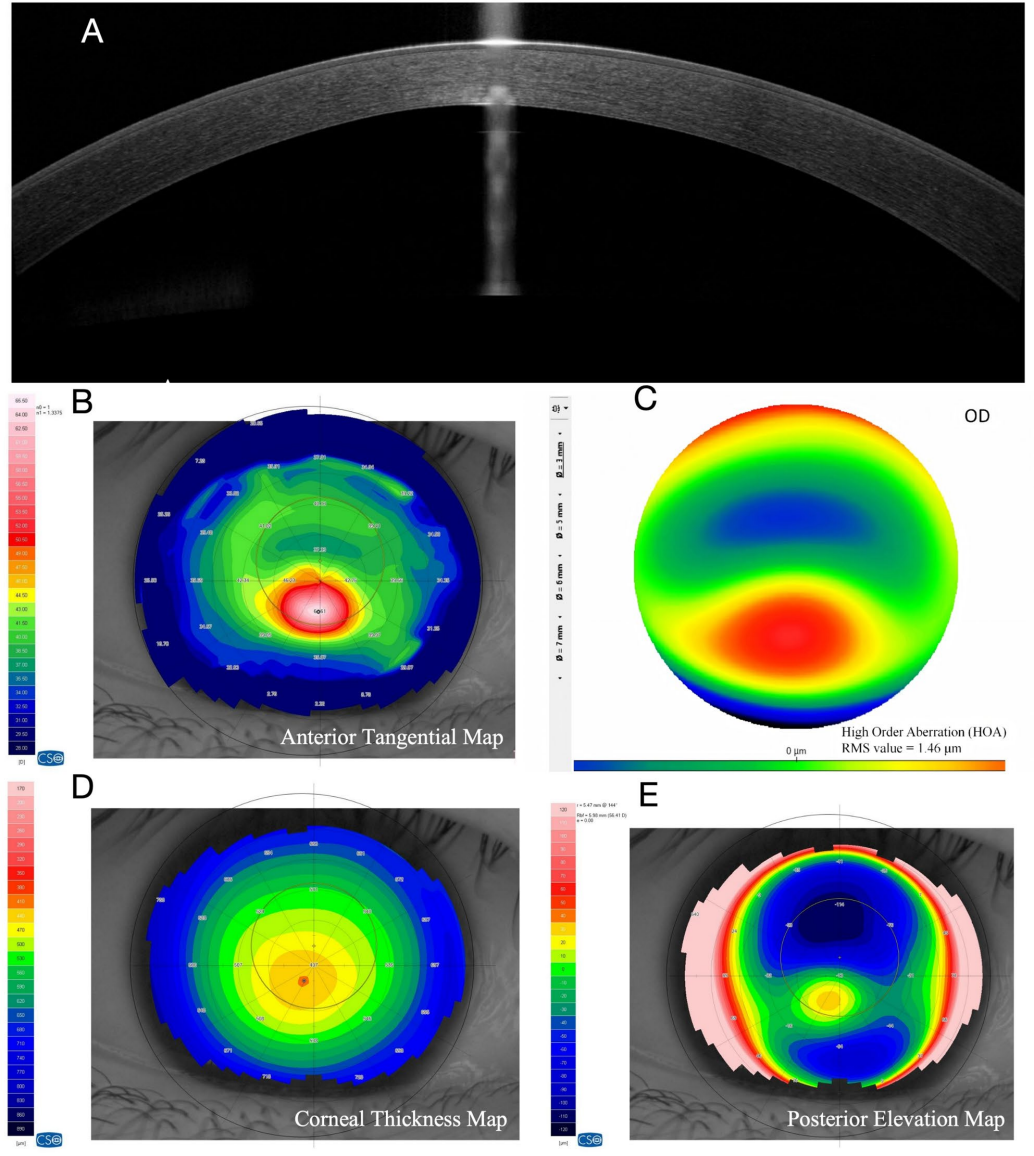
Imaging of a Representative Case without the Halo Sign (Group **A**, HMI = 0). A, AS-OCT shows mild posterior corneal protrusion with preserved stromal architecture. **B**, The tangential curvature map demonstrates localized inferocentral steepening. **C**, The HOA map confirms superior-inferior asymmetry with significant coma. **D**, Corneal pachymetry (thickness) map shows an asymmetric thickness distribution with a decentered thinnest area. **E**, Posterior corneal elevation map shows an asymmetric posterior elevation pattern with a focal decentered elevation area

### Case 4 L: Regular Halo formation in moderate to advanced keratoconus (Group B, HMI = 1.36; Fig. 5)

A 23-year-old male presented with stage 4 keratoconus. The patient reported progressive vision loss in the left eye for over one year. He had a history of wearing frame glasses and had not undergone other correction methods. Examination revealed an uncorrected distance visual acuity (UDVA) of 0.92 in the left eye. Manifest refraction indicated severe compound myopic astigmatism (-5.75 DS / -9.50 DC × 115°), with a corrected distance visual acuity (CDVA) of only 0.80. Slit-lamp biomicroscopy revealed Vogt’s striae and a Fleischer’s ring. Under oblique slit illumination, a complete, regular annular halo was projected onto the iris. AS-OCT demonstrated marked anterior and posterior corneal protrusion and increased stromal reflectivity. Tangential curvature mapping showed a well-defined conical apex with concentric steepening, accompanied by elevated corneal power. The regular halo morphology was consistent with structurally advanced but non-scarred keratoconus.

**Fig. 5:**
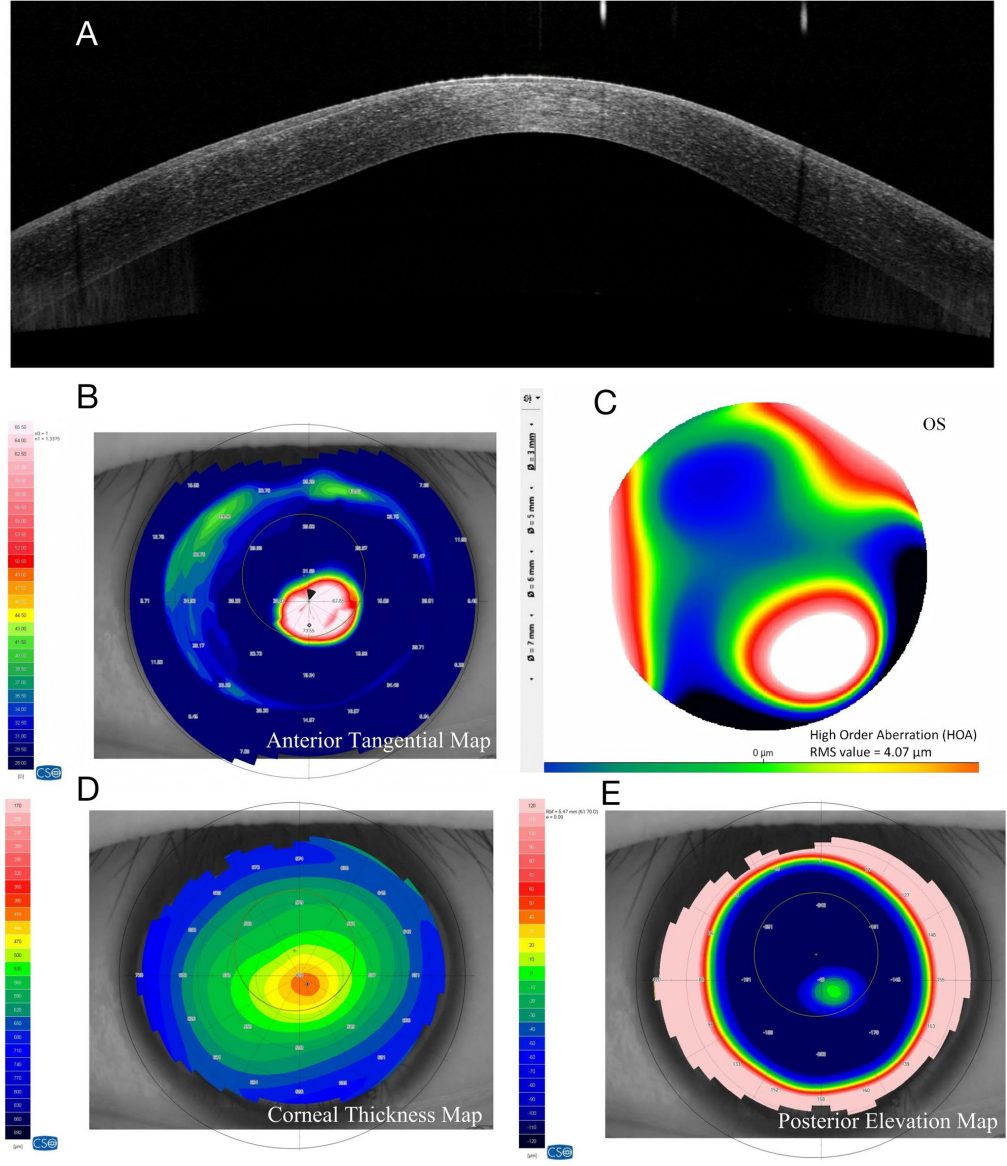
Imaging of a Representative Case with a Regular Halo Sign (Group B, HMI = 1.36). **A**, AS-OCT reveals marked protrusion of both anterior and posterior corneal surfaces and stromal hyperreflectivity in the ectatic area. **B**, The tangential curvature map shows a distinct conical apex with concentric steepening. **C**, The HOA map indicates severe optical irregularity and worsening corneal asymmetry. **D**, Corneal pachymetry (thickness) map shows an asymmetric thickness distribution with a focal thinnest area displaced inferiorly from the corneal center. **E**, Posterior corneal elevation map shows a focal, decentered posterior elevation area located inferiorly

### Case 9 L: Irregular Halo formation in severe keratoconus with scarring (Group B, HMI = 3.53; Fig. 6)

A 19-year-old male presented with stage 4 keratoconus. The patient reported a 1-year history of progressive vision loss in the left eye and admitted to chronic eye rubbing. He was treatment-naive, having never utilized any form of optical correction, including frame glasses. Due to severe corneal optical irregularity, the automatic refractometer could not obtain a reading. Manifest refraction revealed + 1.00 DS, yielding a corrected distance visual acuity (CDVA) of 1.00. Slit-lamp examination revealed Vogt’s striae, a Fleischer’s ring, and stromal scarring at the cone apex. When illuminated, the halo appeared incomplete and fragmented. AS-OCT revealed significant stromal hyper-reflectivity and focal flattening at the apex. The tangential curvature map displayed an expanded and asymmetric steepened zone, and the HOA map demonstrated extensive optical irregularity. The highly distorted halo morphology reflected advanced ectatic deformation.

**Fig. 6:**
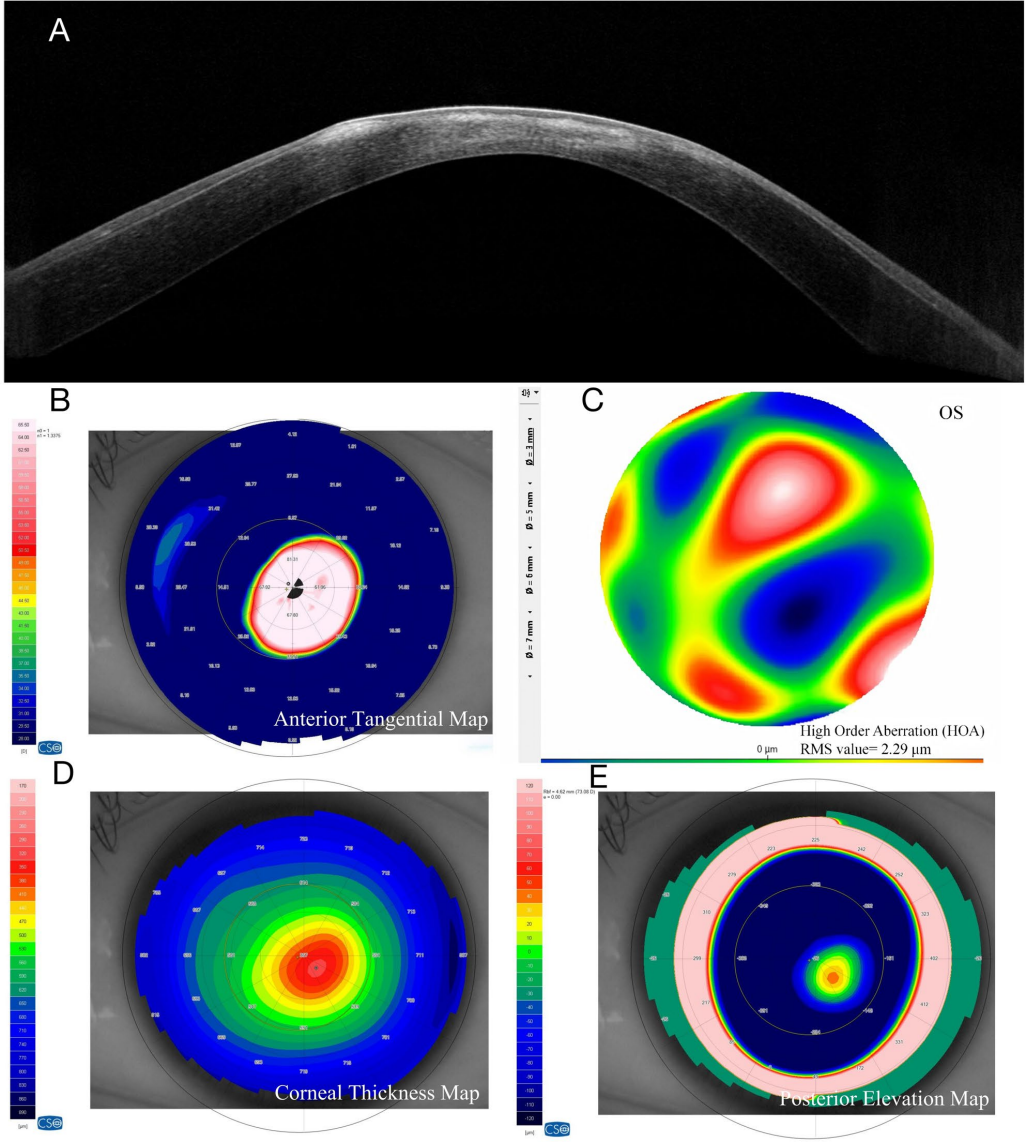
Imaging of a Representative Case with an Irregular Halo Sign (Group B, HMI = 3.53). **A**, AS-OCT demonstrates significant stromal scarring at the cone apex, leading to focal flattening of the corneal surface. **B**, The tangential curvature map shows an expanded area of concentric steepening compared with the case in Fig. 5. **C**, The HOA map illustrates profound optical system irregularity. **D**, The corneal thickness map shows a localized area of thinning with an eccentrically positioned thinnest region. **E**, The posterior corneal elevation map shows a focal posterior elevation pattern corresponding to the ectatic region

## Discussion

KC is a progressive ectatic disorder that predominantly affects adolescents and young adults [1, 22]. Furthermore, the initial responsibility for screening often falls upon primary eye care providers and optometrists using standard equipment [23]. This highlights a critical unmet need to enhance the diagnostic utility of the widely accessible slit-lamp examination by developing quantifiable slit-lamp–derived metrics for KC. While advanced imaging modalities such as corneal tomography are essential for diagnosis and staging, slit-lamp examination remains a fundamental clinical tool for observing secondary morphological and optical manifestations of the disease. Descriptive characterization of slit-lamp findings may enhance understanding of how structural deformation translates into visible optical phenomena. In this context, we investigated the “Halo Sign” as an observational optical manifestation in KC.

In this exploratory, cross-sectional case series, we characterized the Halo Sign as a novel slit-lamp optical phenomenon in keratoconus. Halo-positive eyes were predominantly advanced by clinical staging (all Halo-positive eyes were AK stage IV), whereas Halo-negative eyes spanned a wider range of AK stages. Within Halo-positive eyes, HMI values varied from near-annular to highly anisotropic/fragmented appearances, and these higher HMI values tended to co-occur with more severe tomographic features such as corneal thinning and steepening. These observations are descriptive and hypothesis-generating.

Practical considerations for routine use. In this exploratory case series, the Halo Sign was assessed under slit-lamp biomicroscopy and recorded descriptively based on its presence or absence and overall visible morphology. This qualitative assessment was performed during routine slit-lamp examination. By contrast, quantitative HMI measurement in our study required slit-lamp photography and offline ImageJ analysis, including frame selection, manual ROI delineation of the visible halo envelope, and ellipse fitting to obtain the aspect ratio (HMI = a/b). The analysis time was not prospectively recorded in this retrospective study; however, in our workflow it required offline processing after image acquisition and depended on image quality and halo visibility. In settings where corneal tomography is unavailable, qualitative recognition of the Halo Sign may provide an additional slit-lamp observation that may prompt further evaluation or referral. However, neither Halo Sign presence nor HMI should be used as a stand-alone diagnostic or staging criterion.

In our descriptive data, higher HMI values tended to co-occur with worse CDVA (logMAR), higher AK stage, steeper keratometric indices (e.g., MPP, Km, and thinnest-point curvature), larger KVD, and thinner Thk. This pattern suggests that halo morphology may reflect geometric and structural severity rather than a random optical artifact, although independent contributions of severity, scarring, and decentration cannot be determined in this small cohort. Notably, the Halo Sign was not universally present in advanced disease (a subset of AK stage IV eyes lacked a detectable halo), indicating that it should be considered a complementary observation that may prompt further tomographic evaluation rather than a stand-alone diagnostic criterion.

Compared with Group A, Group B exhibited markedly higher levels of HOAs(1.45–11.93 μm) and coma(0.91–10.05 μm), indicating greater corneal optical irregularity. Interestingly, the values in Group B also demonstrated a broader distribution and increased inter-individual variability, reflecting a wider range of aberration magnitudes among cases. In our cohort, severe keratoconus cases frequently presented with stromal scarring and marked surface irregularity, which may increase variability in aberrometric measurements. Previous studies have highlighted the challenges of accurately reconstructing highly irregular corneal surfaces using standard Zernike polynomials in advanced ectasia [24–26]. HMI may capture light scatter or visibility-related phenomena that are complementary to conventional aberrometric metrics. Therefore, the Halo Sign may serve as a supplementary indicator, particularly in advanced cases with increased measurement variability.

These observations suggest that the Halo Sign may not be a purely incidental optical artifact. However, its precise origin and the mechanisms underlying its morphological variations remain unclear. Multiple pathophysiological factors could provide possible explanations for this. The changes in shape (geometry, such as thickness and curvature) associated with KC are actually manifestations of structural (e.g., collagen fiber organization) and compositional (e.g., proteoglycans, collagen, and keratinocyte quantity) alterations. Alterations in structure or composition typically manifest as changes in corneal shape, mechanical properties, and optical characteristics [27]. When the light source of a slit-lamp microscope is projected onto a keratoconic cornea with significant thickness variations, interference of equal thickness may occur, resulting in redistribution of light intensity [28, 29]. The interference outcome depends on the optical path difference between the two beams [30, 31]. We propose that in less severe cases with a regular halo (e.g., HMI with low AR values), the phenomenon may be primarily driven by the principles of caustics or thin-film interference. The highly curved, thinning cornea acts as an irregular lens, focusing light rays onto a ring-shaped pattern on the iris. The approximately concentric nature of the keratoconic bulge would naturally lead to such an annular pattern. In contrast, the fragmentation and irregularity of the halo in the most advanced cases (e.g., HMI with high AR values) are likely dominated by increased light scattering. As KC progresses, fragmentation of Bowman’s layer and stromal disorganization lead to fibrosis and scarring [1, 32–34], which significantly reduces corneal transparency [35–37]. This increased scattering disrupts the focused light path [38], causing the well-defined halo to degrade into an irregular, fragmented spot, a finding consistent with the presence of corneal scarring in our most severe cases.

Our study has several strengths. To our knowledge, it is the first to systematically describe this novel clinical sign in keratoconus. We introduced a quantitative metric (HMI) to characterize its morphological features and provided a descriptive comparison with established objective indicators of KC severity.

Nonetheless, this study is not without limitations. The primary limitation is the small sample size, which limits generalizability and constrains statistical stability; therefore, findings should be interpreted as exploratory and hypothesis-generating rather than confirmatory. Second, this was a cross-sectional study; therefore, we could not assess the utility of the Halo Sign in monitoring disease progression over time. Future longitudinal studies are required to determine whether within-eye changes in the HMI track documented keratoconus progression. Finally, the HMI is an operational, scale-invariant descriptor of halo outline anisotropy derived from ellipse fitting; it does not directly measure ring completeness (arc-length fraction, continuity, or gap size). Future work should evaluate complementary shape descriptors (e.g., circularity, solidity, and arc-length fraction) under standardized imaging.

In conclusion, the Halo Sign is a newly described slit-lamp optical phenomenon that was observed predominantly in advanced keratoconus in this exploratory case series. Qualitative recognition of the sign at the slit lamp may help prompt further tomographic evaluation, and HMI provides an operational descriptor of halo outline anisotropy for documentation and future research. Larger prospective studies with standardized imaging and prespecified analyses are needed to validate these observations and clarify the roles of severity, scarring, and cone decentration.

## Data Availability

The datasets used and/or analysed during the current study are available from the corresponding author on reasonable request.

## Abbreviations

CDVA: Corrected Distance Visual Acuity
CI: Confidence Interval
HMI: Halo Morphology Index
HOA: Higher-Order Aberrations
KC: Keratoconus
Km: Mean Keratometry
KVD: Keratoconus Vertex Distance
LogMAR: Logarithm of the Minimum Angle of Resolution
MPP: Mean Pupillary Power
SD: Standard Deviation
Thk: Thinnest Corneal Thickness
